# Serum components influence antibody reactivity to glycan and DNA antigens

**DOI:** 10.1038/s41598-023-40707-3

**Published:** 2023-08-22

**Authors:** Tetsuya Okuda, Katsuya Kato

**Affiliations:** 1 Bioproduction Research Institute, National Institute of Advanced Industrial Science and Technology (AIST), Central 6, 1-1-1 Higashi, Tsukuba, 305-8566 Japan; 2 Multi-Material Research Institute, National Institute of Advanced Industrial Science and Technology (AIST), 2266-98 Anagahora, Shimoshidami, Moriyama-ku, Nagoya, 463-8560 Japan

**Keywords:** Biochemistry, Immunology

## Abstract

We previously generated three types of anti-glycan monoclonal IgM antibodies that react with certain structures on the glycans of glycosphingolipids and glycoproteins. As the nucleotide sequences for the variable regions of these IgM antibodies showed homology with those of anti-DNA antibodies deposited in public databases, we analyzed the reactivity of the anti-glycan IgM antibodies to DNA by ELISA. We found that anti-α2,6-sialyl LacNAc IgM in the supernatant of a hybridoma culture cross-reacted with DNA, and after purification of the IgM by zirconia column chromatography, the highly purified IgM showed increased cross-reactivity to DNA. As most of the contaminating bovine serum proteins in the culture supernatant were removed by the purification process, it is likely that a part of the removed components influences antibody reactivity to DNA. Purified anti-DNA antibodies prepared from lupus model NZB/W F1 and MRL/lpr mouse sera and normal human serum were then analyzed, and similar results showing increased reactivity to DNA were obtained. Furthermore, ELISA using these purified antibodies and various carbohydrate antigens showed that the antigen-binding specificity of these antibodies was altered by the purification process from serum-containing antibody preparations. Our results indicate that mammalian serum contains components that strongly influence antibody reactivity to carbohydrate antigens, including DNA.

## Introduction

Human blood contains antibodies that recognize specific structures on the glycans of glycoproteins and glycosphingolipids. These antibodies in human blood have useful medical applications, e.g., antibodies that recognize ABO blood group glycan antigens are used for the matching of blood types in blood transfusion^1,2^, and anti-glycosphingolipid antibodies are used for the diagnosis and evaluation of autoimmune diseases, such as Guillain-Barré syndrome, as they increase with disease progression^2,3^. Antibodies that react with nucleic acids have also been well studied as serum antibodies that serve as diagnostic indicators in autoimmune diseases^4^. In particular, in systemic lupus erythematosus (SLE), antibodies that recognize single-stranded DNA (ssDNA) or double-stranded DNA (dsDNA) are used as diagnostic indicators that reflect disease progression^4,5^. The levels of autoimmune disease-related antibodies in blood are known to change with disease progression. It has also been speculated that these antibodies influence the pathology of different autoimmune diseases, although no direct evidence has yet been shown.

It has also been reported that various glycan antigens reflect the progression of the cellular pathology of human diseases, such as cancer, and infectious and inflammatory diseases^2,6,7^. As antibodies that specifically react with specific glycan structures on the cell surface are useful for the diagnosis and treatment of these diseases, we have been developing technology for efficiently obtaining anti-glycan monoclonal antibodies^7–10^. Using this technology, we previously established three types of hybridoma cells, FR9, AFR45, and PA5 cells, which produce immunoglobulin M (IgM) antibodies recognizing certain glycan structures as epitopes^8,11–13^, and determined the nucleotide sequences for their heavy and light chain variable regions (V_H_ and V_L_, respectively)^10,11^. Hybridoma FR9 cells produce IgM that specifically recognizes the α2,6-sialyl LacNAc structure of glycans on glycoproteins and glycosphingolipids^8,11^. Hybridoma AFR45 cells produce IgM that recognizes α2,3-sialylated glycans, such as GM3 ganglioside^11^. Hybridoma PA5 cells produce IgM that specifically recognizes the glycan structure of glycosphingolipid globoside (Gb4)/P antigen^10,12,13^.

In the present study, we analyzed the nucleotide sequences for the V_H_ and V_L_ regions of the IgM antibodies from the FR9, AFR45, and PA5 cells (FR9 IgM, AFR45 IgM, and PA5 IgM, respectively; the anti-glycan IgM antibodies), and examined by enzyme-linked immunosorbent assay (ELISA) the DNA-binding properties of these IgM antibodies before and after purification by zirconia column chromatography.

## Results

### Comparative analysis of the variable regions of immunoglobulins recognizing glycans and DNA

The results of a Basic Local Alignment Search Tool (BLAST) search using the nucleotide sequences for the V_H_ and V_L_ regions of the anti-glycan IgM antibodies revealed high homology with several anti-DNA antibodies deposited in public databases (Supplementary Table S1). Most of these anti-DNA antibodies were monoclonal antibodies isolated from lupus model mice, such as NZB/W F1 and MRL/lpr mice^14–16^, and several classes of antibodies included not only IgM, but also immunoglobulin G (IgG). Although homology was not seen in both the V_L_ and V_H_ regions between any of the anti-glycan IgM antibodies and anti-DNA antibodies, high homology (over 94%) was seen between several of the nucleotide sequences for either the V_H_ or V_L_ region. Differences were found mainly in the D and J gene segments approximately 300 bp downstream of the variable regions (Supplementary Fig. S1).

### Analysis of the reactivity of anti-glycan IgM antibodies to various glycan antigens and DNA

**Figure 1:**
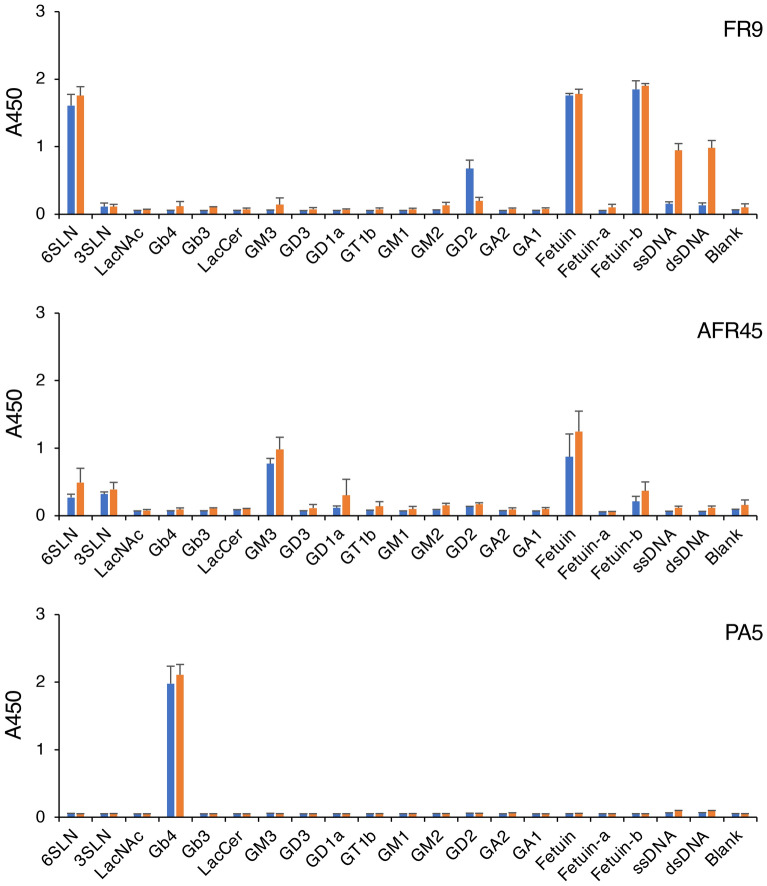
Reactivity of anti-glycan IgM to various carbohydrate antigens. Hybridoma culture supernatants containing anti-glycan IgM (blue bars) or IgM purified from the supernatant by zirconia column chromatography (orange bars) were incubated in glycoconjugate- or DNA-coated microplate wells, and the reactivity of each IgM sample to the well was detected by ELISA. Top panel, FR9 IgM reactivity; middle panel, AFR45 IgM reactivity; bottom panel, PA5 IgM reactivity. In this assay, each IgM was used at a final concentration of 2 µg/ml. Error bars indicate the mean ± standard deviation (n = 4). The structures of the glycoconjugates used in this analysis are shown in Supplementary Table S2.

**Figure 2:**
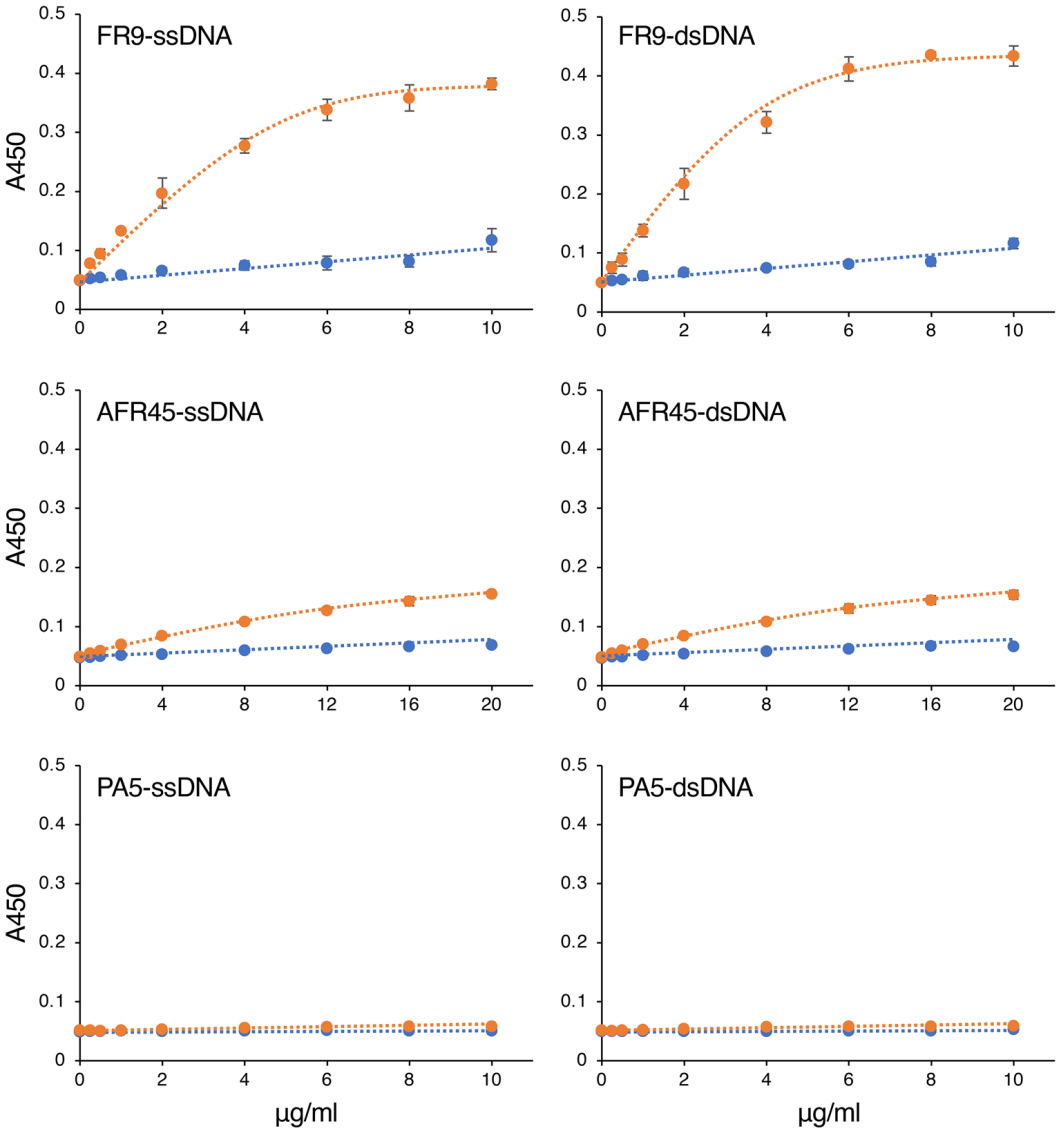
Concentration-dependent reaction of anti-glycan IgM to DNA. Serially diluted hybridoma culture supernatants containing anti-glycan IgM (blue lines) or serially diluted IgM antibodies purified from the supernatant by zirconia column chromatography (orange lines) were incubated in ssDNA-coated (left panels) or dsDNA-coated (right panels) microplate wells, and the reactivity of each IgM sample to the well was detected by ELISA. The IgM concentration of each sample is indicated on the x-axis. Top panel, FR9 IgM reactivity; middle panel, AFR45 IgM reactivity; bottom panel, PA5 IgM reactivity. Error bars indicate the mean ± standard deviation (n = 4).

To investigate whether the anti-glycan IgM antibodies cross-react with DNA, we analyzed their reactivity to ssDNA and dsDNA. Various glycan antigens were also used in this study for comparison. First, we performed ELISA using the hybridoma culture supernatant containing the anti-glycan IgM antibodies, which acted as the primary antibodies (Fig. 1, blue bars); only FR9 IgM showed a weak antibody reaction to the DNA (Fig. 1, FR9). To examine this reaction in more detail, we then performed ELISA using purified IgM prepared by a zirconia column chromatography method optimized for purifying IgM and IgG^17,18^. Surprisingly, the ELISA results showed that the signal from FR9 IgM reacting to DNA increased approximately sevenfold after the purification process. We also performed ELISA using different concentrations of FR9 IgM, and an antibody concentration-dependent increase in the reaction signals was detected (Fig. 2, orange line in FR9-ssDNA and FR9-dsDNA). The signal intensity was similar when ssDNA and dsDNA were used as ELISA antigens, indicating that FR9 IgM binds to structures that are in common between ssDNA and dsDNA. In contrast, when unpurified antibody was used, only weak signals were detected, even when a high concentration of IgM antibody was used (Fig. 2, blue line in FR9-ssDNA and FR9-dsDNA); this suggested that the reaction was inhibited by the components that were removed during zirconia chromatography purification. We further analyzed whether DNA in the nucleus of methanol-fixed HeLa cells could be stained with the purified FR9 IgM, but no signal was detected. As FR9 is an IgM class antibody, it does not appear to be suitable for DNA staining in the nucleus.

ELISA using AFR45 IgM also revealed slight reaction signals (Fig. 2, orange lines in AFR45-ssDNA and AFR45-dsDNA), but the intensities were low, and were detected only when high concentrations of purified AFR45 IgM were used; this suggested that the signals were from non-specific binding. In contrast, PA5 IgM specifically binds to Gb4 but was completely unreactive with other ELISA antigens, including DNA (Figs. 1 and 2, PA5).

### Analysis of the contaminants in the IgM solutions purified by zirconia chromatography

**Figure 3:**
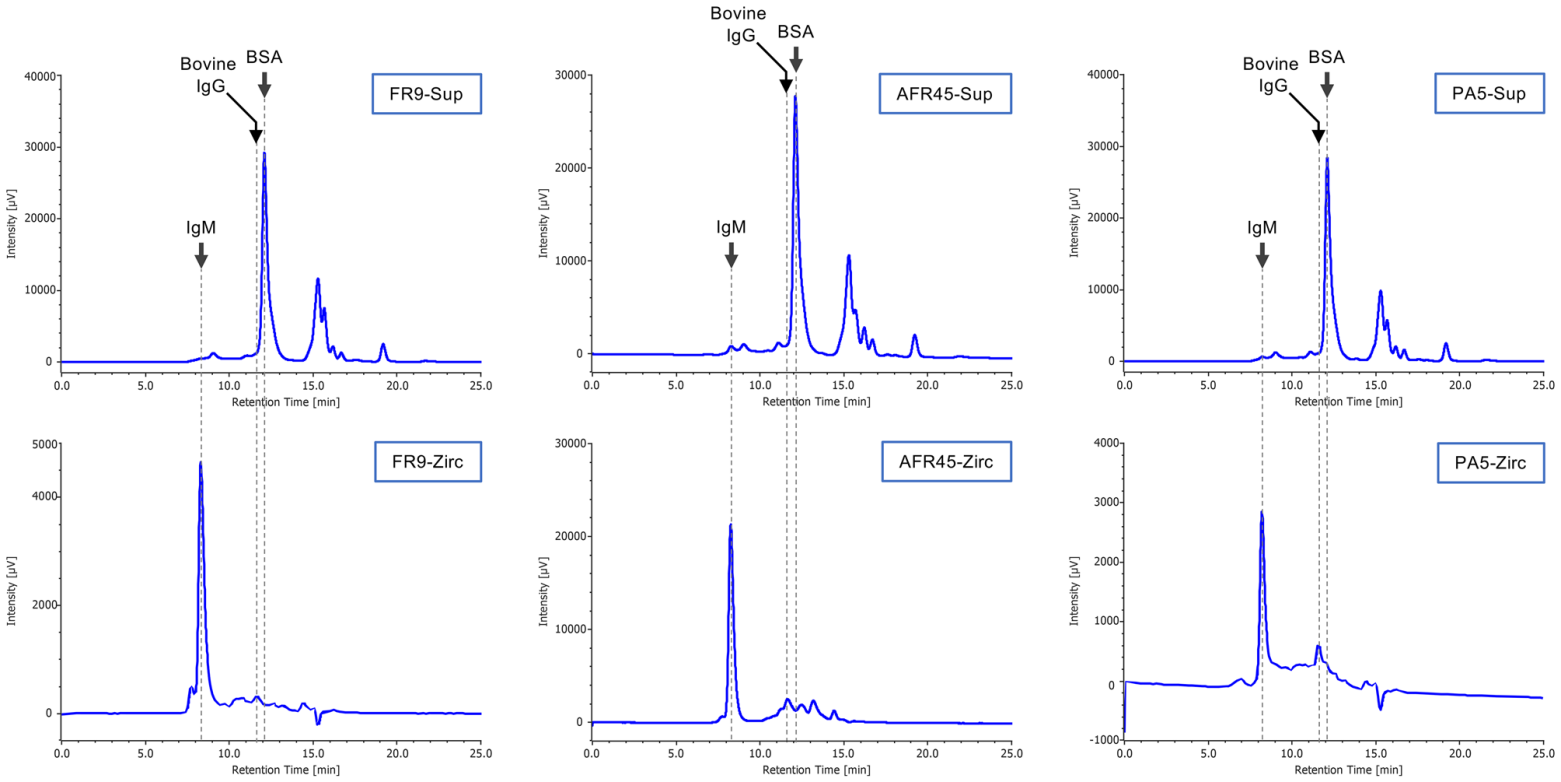
SEC-HPLC analysis of IgM samples. IgM antibodies in the culture supernatants of FR9, AFR45, and PA5 hybridomas were purified by zirconia column chromatography, and 10 μl of each supernatant or purified IgM was analyzed by SEC-HPLC. *FR9-Sup* culture supernatant of the FR9 hybridoma, *AFR45-Sup* culture supernatant of the AFR45 hybridoma, *PA5-Sup* culture supernatant of the PA5 hybridoma, *FR9-Zirc* zirconia-purified FR9 IgM, *AFR45-Zirc* zirconia-purified AFR45 IgM, *PA5-Zirc* zirconia-purified PA5 IgM. Arrows indicate the retention times of the standard mouse IgM, bovine IgG, and BSA as references.

To analyze the changes in the components in the IgM solutions after purification by zirconia chromatography, the contaminating proteins in the purified and unpurified IgM solutions were analyzed by size exclusion-high-performance liquid chromatography (SEC-HPLC). As the unpurified IgM solutions were the culture supernatant of each hybridoma, the solutions contained a large amount of bovine serum proteins, such as bovine serum albumin (BSA)^17,18^. In the solutions, IgM was a minor protein component, and was barely detectable (Fig. 3, FR9-Sup, AFR45-Sup, and PA5-Sup), but after purification by zirconia chromatography, IgM became the major protein component (Fig. 3, FR9-Zirc, AFR45-Zirc, and PA5-Zirc). In all cases, the purification of IgM from hybridoma culture supernatants by zirconia chromatography resulted in highly pure IgM preparations. As the zirconia column used in this purification method can selectively adsorb and purify various immunoglobulins, a weak peak from bovine serum-derived IgG was also detected in the purified IgM solution.

### Effect of purification by zirconia chromatography on the reactivity of anti-DNA antibodies prepared from lupus model mice

**Figure 4:**
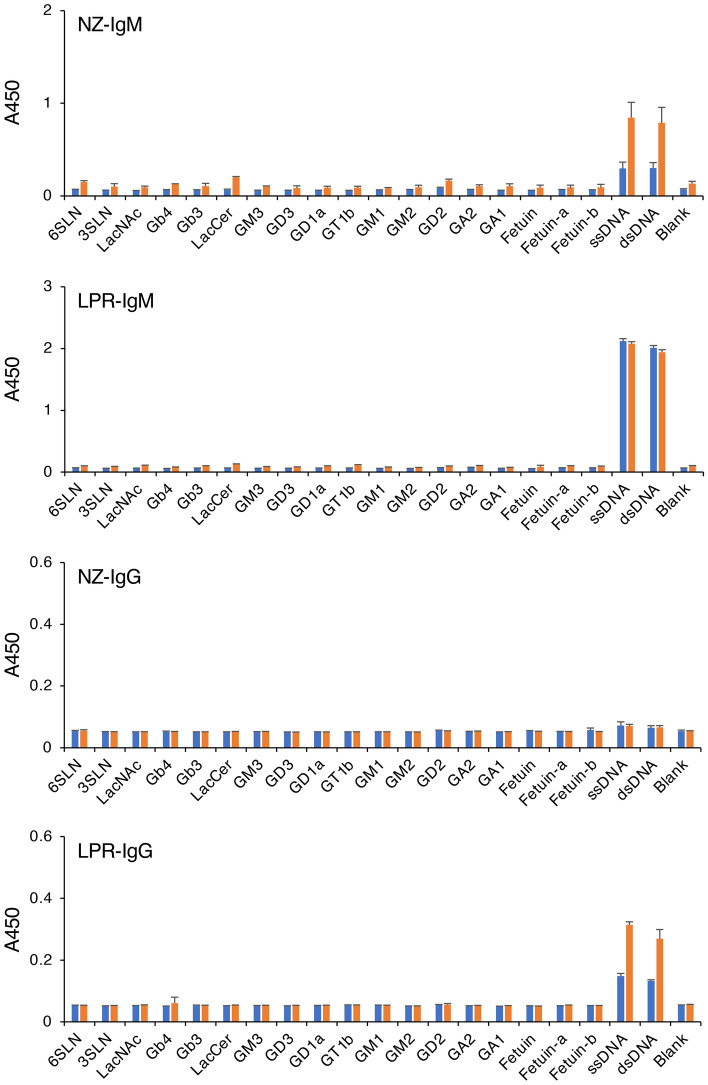
Reactivity of immunoglobulins in lupus model mouse serum to various carbohydrate antigens. Serum samples (blue bars) or immunoglobulins purified from serum by zirconia column chromatography (orange bars) were incubated in glycoconjugate- or DNA-coated microplate wells, and the reactivity of the IgM or IgG antibodies in each sample to the well was detected by ELISA. *NZ-IgM* IgM antibodies in NZB/W F1 mouse serum, *LPR-IgM* IgM antibodies in MRL/lpr mouse serum, *NZ-IgG* IgG antibodies in NZB/W F1 mouse serum; *LPR-IgG* IgG antibodies in MRL/lpr mouse serum. In this assay, each IgM was used at a final concentration of 2 µg/ml. Error bars indicate the mean ± standard deviation (n = 4).

**Figure 5:**
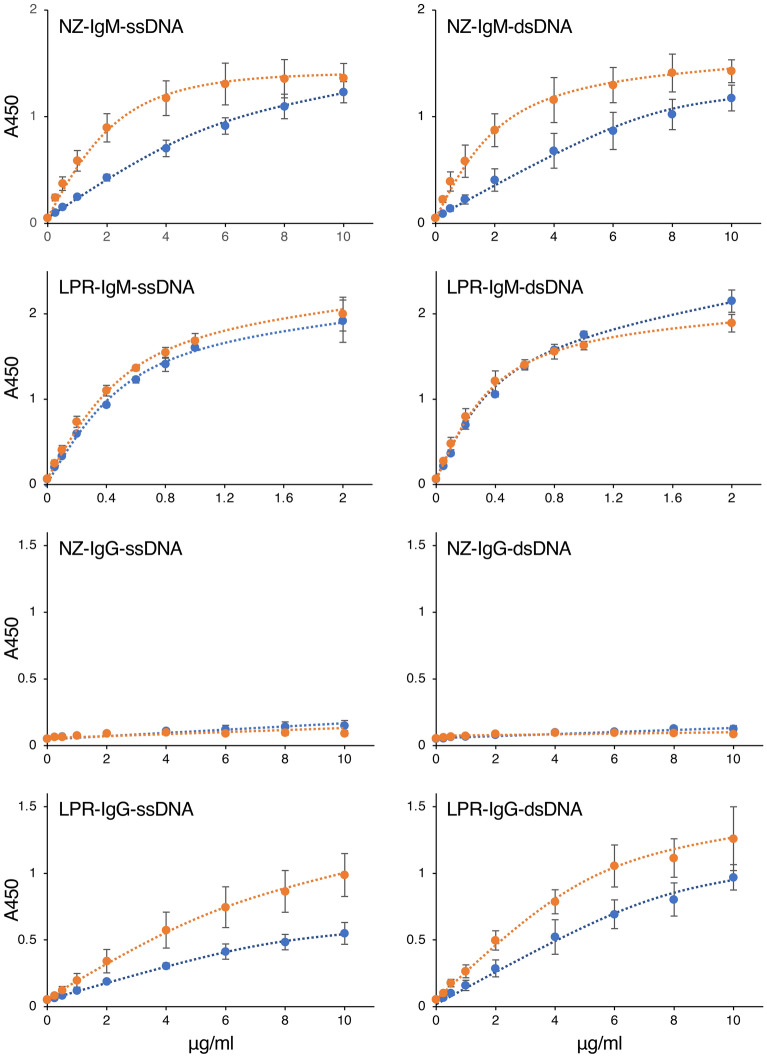
Concentration-dependent reaction of the immunoglobulins in lupus model mouse serum to DNA. Serially diluted serum sample (blue lines) or serially diluted immunoglobulins purified from the serum by zirconia column chromatography (orange lines) were incubated in ssDNA-coated (left panels) or dsDNA-coated (right panels) microplate wells, and the reactivity of the IgM or IgG antibodies in each sample to the well was detected by ELISA. The immunoglobulin concentration of each sample is indicated on the x-axis. *NZ-IgM* IgM antibodies in NZB/W F1 mouse serum, *LPR-IgM* IgM antibodies in MRL/lpr mouse serum, *NZ-IgG* IgG antibodies in NZB/W F1 mouse serum, *LPR-IgG* IgG antibodies in MRL/lpr mouse serum. Error bars indicate the mean ± standard deviation (n = 4).

The ELISA and SEC-HPLC results of the purified FR9 IgM indicated that the serum contained components that inhibit the binding of FR9 IgM to DNA. To investigate whether serum contains components that can also inhibit the binding of autoimmune disease-related anti-DNA antibodies to DNA, we investigated the anti-DNA reactivity of serum antibodies prepared from two strains of SLE model mice before and after purification by zirconia chromatography. In this experiment, we used sera prepared from 7- to 8-week-old female lupus model NZB/W F1 and MRL/lpr mice^14–16^. The ELISA results showed anti-DNA reactivity from the IgM in the NZB/W F1 mouse serum, and the IgM and IgG antibodies in the MRL/lpr mouse serum (Fig. 4, NZ-IgM, LPR-IgM, and LPR-IgG). These reactions were detected at a similar signal intensity even when ssDNA and dsDNA were used as the ELISA antigens, indicating that these immunoglobulins recognize structures that are in common between ssDNA and dsDNA. Among these serum antibodies, the DNA-binding signals of IgM antibodies in NZB/W F1 mouse serum and IgG antibodies in MRL/lpr mouse serum were clearly increased by the zirconia purification process (Fig. 4, orange bars in NZ-IgM and LPR-IgG). The antibody reactions were also increased in an antibody concentration-dependent manner (Fig. 5, NZ-IgM and LPR-IgG), indicating that the reactions were DNA-specific antigen–antibody reactions. In contrast, the anti-DNA reactivity of IgM antibodies in MRL/lpr mouse serum remained unchanged before and after the purification (Figs. 4 and 5, LPR-IgM); the DNA-binding signals were very strong, and were clearly detected even when a low concentration of IgM was used in the ELISA. These results indicated that the mouse sera also contained components that inhibit the binding of antibodies to DNA, but the effect was negligible when the antibodies bind to DNA with high affinity.

**Figure 6:**
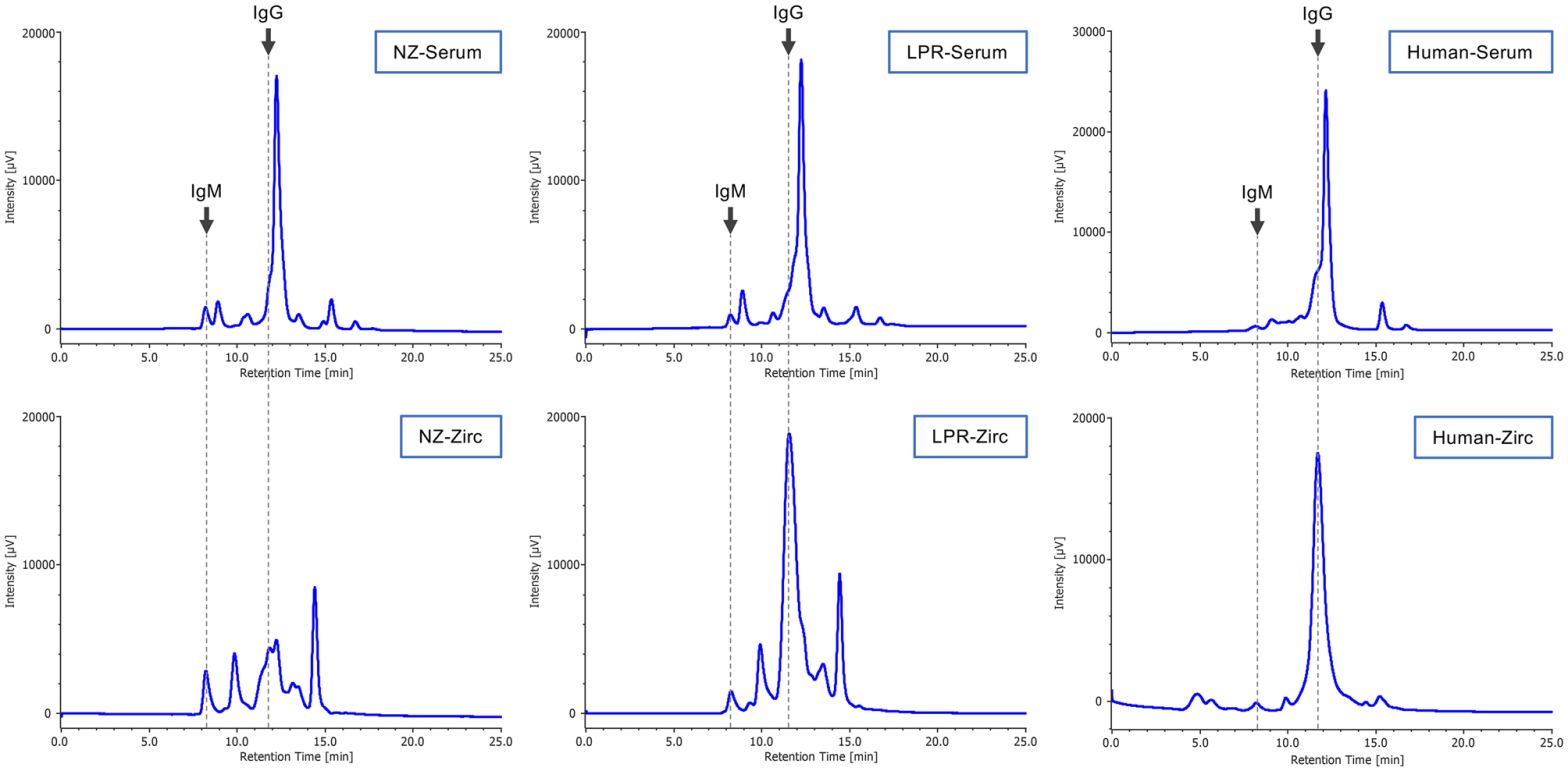
SEC-HPLC analysis of immunoglobulins in lupus model mice and human sera. Immunoglobulins in NZB/W F1 and MRL/lpr mouse sera and a normal human serum were purified by zirconia column chromatography, and 10 μl of diluted serum or purified IgM antibodies were analyzed by SEC-HPLC. *NZ-serum* NZB/W F1 serum, *LPR-serum* MRL/lpr mouse serum, *Human-serum* human serum, *NZ-Zirc* zirconia-purified NZB/W F1 serum, *LPR-Zirc* zirconia-purified MRL/lpr mouse serum, *Human-Zirc* zirconia-purified human serum. Arrows indicate the retention times of the standard IgM and IgG as references.

Further SEC-HPLC analysis showed that the SEC-HPLC patterns differed between the purified mouse immunoglobulin solutions and the IgM solutions purified from hybridoma culture supernatants (Fig. 6, left and middle panels). The main difference was that both IgM and IgG were detected as major peaks in the zirconia-purified mouse sera preparations, because the sera contained both IgM and IgG whereas only IgM was detected as a major peak in the solutions purified from hybridoma culture supernatants (Fig. 6, NZ-Zirc and LPR-Zirc). In addition, while most contaminating proteins were removed, several contaminating proteins became enriched and were detected as peaks. These results indicated that certain components present in animal sera have moderate inhibitory activity on the binding of antibodies to DNA.

### Effect of zirconia purification on the reactivity of anti-DNA antibodies in human serum

**Figure 7:**
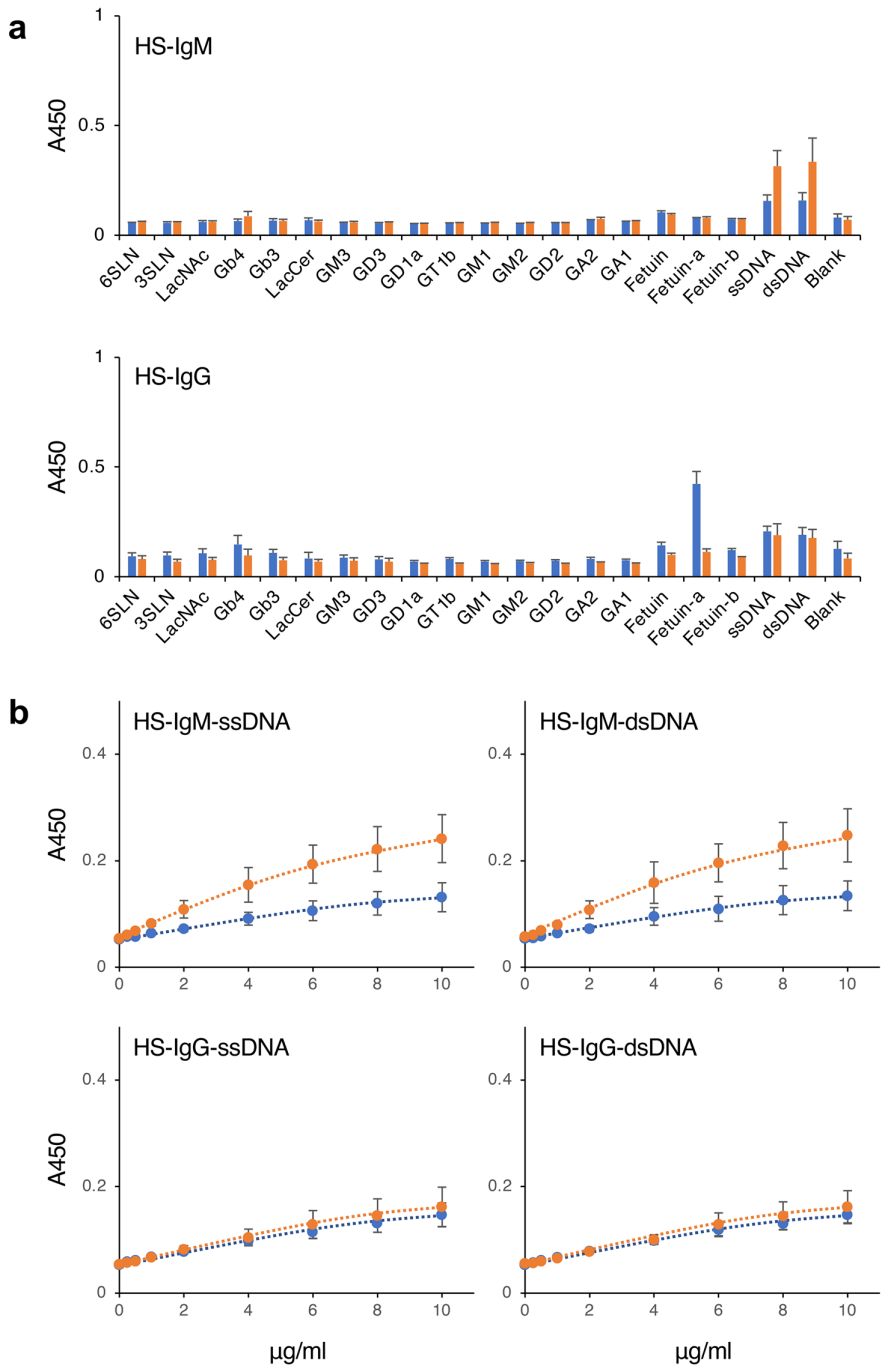
Reactivity of immunoglobulins in human serum to various carbohydrate antigens. (**a**) Serum sample (blue bars) or immunoglobulins purified from the serum by zirconia column chromatography (orange bars) were incubated in glycoconjugate-coated or DNA-coated microplate wells, and the reactivity of the IgM or IgG antibodies in each sample to the well was detected by ELISA. In this assay, each immunoglobulin was used at a final concentration of 2 µg/ml. (**b**) Serially diluted serum sample (blue bars) or serially diluted immunoglobulins purified from the serum by zirconia column chromatography (orange bars) were incubated in ssDNA-coated (left panels) or dsDNA-coated (right panels) microplate wells, and the reactivity of the IgM or IgG antibodies in each sample to the well was detected by ELISA. The immunoglobulin concentration of each sample is indicated on the x-axis. *HS-IgM* IgM antibodies in human serum, *HS-IgG* IgG antibodies in human serum. Error bars indicate the mean ± standard deviation (n = 4).

To investigate whether a similar inhibitory effect on antibody-DNA binding is observed with human serum components, we analyzed the antibody reactivity to DNA of serum antibodies prepared from healthy donors. It has been reported that low-affinity anti-DNA IgM antibodies are present in healthy individuals^5^; indeed, we detected low-affinity anti-DNA IgM antibodies in the human serum (Fig. 7a, HS-IgM). When the human serum was directly used in the ELISA, no clear antibody reaction to DNA was detected. However, after purification of the IgM in the human serum by zirconia chromatography, we detected clear antibody reactions to both ssDNA and dsDNA. The binding signals of the purified human IgM antibodies to ssDNA and dsDNA increased in an antibody concentration-dependent manner (Fig. 7b, HS-IgM), indicating that the antibody reaction was also a DNA-specific antigen–antibody reaction. SEC-HPLC analysis of the purified human immunoglobulin solutions showed that the major contaminating proteins were removed, and immunoglobulins, particularly IgG, were detected as the main peaks after zirconia purification (Fig. 6, right panels). IgM was detected as a small peak by SEC-HPLC, but a constant amount of IgM in the purified IgM solutions was detected using an IgM quantification kit. It is possible that the conformation of the IgM in the samples was not maintained, making it difficult to detect by SEC-HPLC. These results indicated that certain components present in human serum also have moderate inhibitory activity on the binding of antibodies to DNA.

### Effect of zirconia purification on the reactivity of antibodies to various glycan antigens

**Figure 8:**
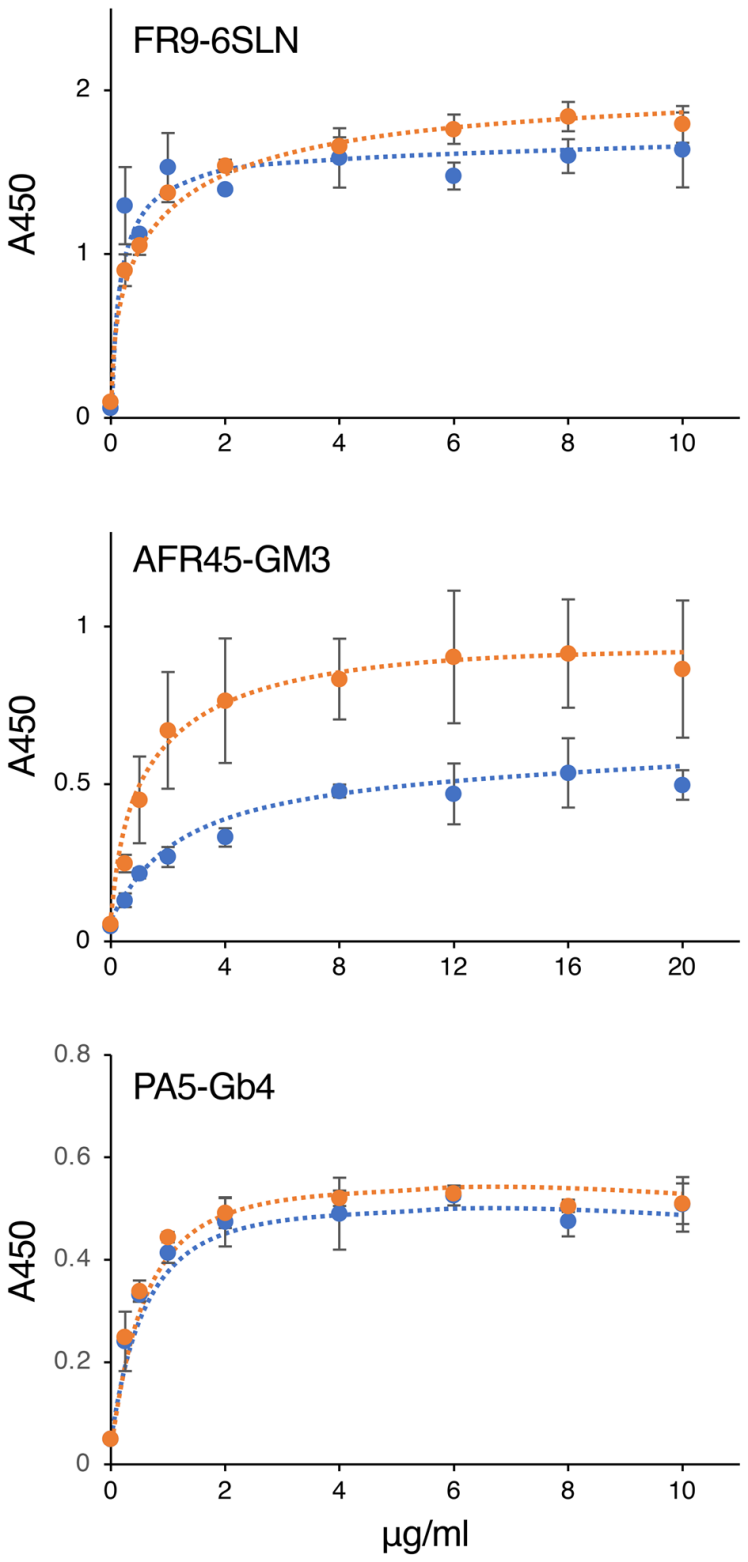
Concentration-dependent reaction of anti-glycan IgM antibodies to their glycan epitopes. Serially diluted hybridoma culture supernatant containing anti-glycan IgM (blue lines) or serially diluted IgM antibodies purified from the supernatant by zirconia column chromatography (orange lines) were incubated in glycan epitope-coated microplate wells, and the reactivity of each IgM sample to the well was detected by ELISA. The IgM concentration of each sample is indicated on the x-axis. FR9-6SLN, reaction of FR9 IgM to 6SLN; AFR45-GM3, reaction of AFR45 IgM to GM3; and PA5-Gb4, reaction of PA5 IgM to Gb4. Error bars indicate the mean ± standard deviation (n = 4). The structures of 6SLN, GM3, and Gb4 are shown in Supplementary Table S2.

The ELISA results showed that the reactivity against glycan antigens of the antibodies analyzed in this study was altered by zirconia purification (Figs. 1, 4, 7, and 8). Similar to the ELISA results for other DNA-binding antibodies, ELISA using AFR45 IgM showed a concentration-dependent increase in the reactivity to GM3, the major epitope of AFR45 IgM, after purification (Fig. 8, AFR45-GM3). This result also indicated that certain components present in bovine serum have inhibitory activity on the binding of AFR45 IgM to GM3.

We previously reported that FR9 IgM specifically reacts with the α2,6-sialyl LacNAc structure found in glycolipids and glycoproteins, such as fetuin^8^. In the present study, we found a new signal from the binding of unpurified FR9 IgM to GD2 ganglioside-coated plates (Fig. 1, FR9). However, this signal was not detected in the ELISA using purified FR9 IgM. ELISA using human serum revealed the reactivity of IgG antibodies in the serum to asialofetuin, but this signal was not detected when using IgG antibodies purified from the human serum (Fig. 7a, HS-IgG, Fetuin-a). These results indicated that these reactions to GD2 and asialofetuin were non-specific reactions of IgM and IgG antibodies mediated by the contaminants that were removed from the bovine or human serum by zirconia purification.

## Discussion

The nucleotide sequences for the variable regions of the anti-glycan IgM antibodies examined in this study showed high similarity to those of anti-DNA antibodies isolated from lupus model mice. Among the examined IgM antibodies, FR9 IgM, which recognizes α2,6-sialylated glycans, was found to cross-react with DNA. The signal intensity of the reaction was similar when ssDNA and dsDNA were used as ELISA antigens. This indicated that FR9 IgM binds to sites that are structurally common to ssDNA and dsDNA, such as outer phosphorylated deoxyribose. Phosphorylated deoxyribose and sialylated glycans are similar molecules in that they are negatively charged acidic carbohydrates. Our results indicate that mammals have a set of genes for antibody variable regions that recognize these acidic carbohydrates.

As the antigens recognized by IgM, i.e., glycoconjugates and DNA, are also present in the host mice used to generate the IgM antibodies, these IgM antibodies are autoreactive antibodies. We previously reported that the V_L_ region gene sequences are highly homologous between FR9 and AFR45 IgM antibodies and antibodies that recognize *Neisseria meningitidis* group C capsular polysaccharide^11^. These findings support the concept of “molecular mimicry”, in which carbohydrate antigens derived from microorganisms that infect mammals induce autoreactive antibodies in the host body^19^. Thus, we speculate that these anti-glycan antibodies are induced by pathogenic microbial infections, and are involved in host defense in mammals.

The anti-glycan IgM antibodies used in this study share the common feature of recognizing glycoconjugates expressed on the surface of human umbilical vein endothelial cells (HUVECs; Supplementary Fig. S2); these glycoconjugates are known to be involved in the inflammatory response of vascular endothelial cells^20–25^. However, some patients with SLE suffer from vasculitis, which is characterized by the deposition of immunoglobulins and complement in the walls of blood vessels^4,26^. Thus, anti-glycan IgM-mediated complement deposition on the vascular endothelium may contribute to the inflammatory pathology of autoimmune diseases.

In the present study, the results indicated the possibility that among the anti-DNA antibodies produced by patients with autoimmune diseases, such as SLE, there are antibodies that cross-react with sialylated glycans. Based on this, we additionally analyzed the cross-reactivity to glycan antigens of anti-DNA antibodies prepared from lupus model mice and human serum, but we detected no cross-reactivity with these serum antibodies (Fig. 4  and 7a). At this time, we conclude that the cross-reactivity of anti-α2,6-sialyl LacNAc IgM to DNA is an intrinsic property of this IgM, but further studies may reveal an association between anti-glycan antibodies and the pathogenesis of SLE.

Removal of contaminating serum components from the antibody solutions by zirconia purification increased the reactivity of the antibodies to DNA. In addition, although the IgM antibodies used in this study are autoreactive antibodies that can recognize glycan epitopes present in the host mice, the host mice did not show any symptoms, even after antibody induction. Our results indicate the presence of serum factors that decrease the autoreactivity of these anti-glycan IgM antibodies. As a candidate factor, we focused on fetuin, a major ligand of FR9 IgM, contained in the fetal bovine serum^27^ used in the hybridoma culture medium, but no inhibitory effect on the binding of these antibodies to DNA was observed in a competitive ELISA using fetuin (Supplementary Fig. S3). We also analyzed the effect of free DNA in the antibody solution on the binding of FR9 antibody to DNA by a competitive ELISA using dsDNA (Supplementary Fig. S4), but no inhibitory effect was observed in this competitive ELISA either. In addition to fetuin, many other negatively charged molecules such as acidic proteins and sialylated glycoconjugates are present in serum. All or part of them may cooperatively inhibit antibody binding to sialylated glycans and DNA.

We also observed that removal of the contaminants by zirconia column chromatography purification decreased the non-specific binding of these antibodies to glycan antigens. As this purification method is simple to perform and is effective for removing serum contaminants, it can be applied to improve diagnostic and pathological analyses for various autoimmune diseases related to anti-glycan and anti-DNA antibodies.

## Methods

### Materials

Culture supernatants containing IgM antibodies were prepared from hybridomas maintained in hypoxanthine and thymidine medium (RPMI-1640 culture medium containing 10% fetal bovine serum (Cytiva, Tokyo, Japan), 100 µM sodium hypoxanthine, 16 µM thymidine, and 10 µg/ml gentamicin) at 37 °C in a humidified atmosphere containing 5% CO_2_. The amount of IgM in each sample was measured using an IgM ELISA kit (Thermo Fisher Scientific, Waltham, MA, USA) according to the manufacturer’s protocol. Zirconia particles, i.e., Rhinophase-AB and porous zirconia particles were obtained from ZirChrom Separations (Anoka, MN, USA) and NGK Spark Plug (Aichi, Japan), respectively. Globoside from human erythrocytes and fetuin from fetal calf serum were obtained from Sigma-Aldrich (St. Louis, MO, USA). GM3 (d18:1, C18:0) was obtained from Tokyo Chemical Industry (Tokyo, Japan). Other glycoconjugate standards were obtained or prepared as described previously^17^. DNA from salmon sperm was obtained from Wako (Osaka, Japan). Immunoglobulin standards were obtained from BioLegend (San Diego, CA, USA) and Medical & Biological Laboratories (Tokyo, Japan). BSA was obtained from Nacalai Tesque (Kyoto, Japan). Human serum obtained from a variety of blood types and different sexes was obtained from Biowest (Nuaillé, France); this human serum was collected or imported and treated in accordance with European regulations.

### Comparative analysis of the variable region sequences of immunoglobulins

Homology searching of target gene sequences against the gene sequences deposited in public databases was performed using BLAST (https://blast.ncbi.nlm.nih.gov/Blast.cgi). Homology analysis of nucleotide and amino acid sequences of target genes was performed using GENETYX-MAC (GENETYX, Tokyo, Japan). The nucleotide sequences reported in this paper have been deposited in GenBank under accession numbers LC635723 (V_H_ of FR9), LC635724 (V_L_ of FR9), LC635721 (V_H_ of AFR45), LC635722 (V_L_ of AFR45), LC730313 (V_H_ of PA5), and LC730314 (V_L_ of PA5).

### Preparation of serum

Lupus model NZB/W F1 and MRL/lpr mice were obtained from Japan SLC (Shizuoka, Japan). Mice were housed in a controlled SPF animal room with a temperature of 23 °C ± 2 °C, a humidity of 55% ± 15%, a 12 h light/12 h dark cycle, and were allowed food and water ad libitum. Whole blood was collected from the heart of 7- to 8-week-old mice, and the blood samples from 10 mice were pooled. After incubating the collected blood at 37 °C for 90 min, the clots were removed, and the sample was centrifuged at 3000 rpm for 15 min to collect the supernatant as serum. The Committee for Experiments Involving Animals of the National Institute of Advanced Industrial Science and Technology (AIST) approved all animal experiments. All experiments were performed in accordance with the relevant guidelines and regulations and reported in accordance with ARRIVE guidelines^28^.

### Zirconia column chromatography

Purification of antibodies using zirconia column chromatography was performed as described previously^18^. The hybridoma culture supernatant and serum were diluted threefold and 30-fold, respectively, with 10 mM phosphate buffer (pH 7.0). The diluted solution was passed through a column packed with 200 μl of zirconia particles at a flow rate of 1.5 ml/min to adsorb the immunoglobulins. After running the sample, the column was washed with 4 ml of 10 mM phosphate buffer (pH 7.0), and the adsorbed immunoglobulins were eluted with 1 ml of 400 mM phosphate buffer (pH 8.0). Rhinophase-AB was mainly used for the zirconia column in this experiment; since PA5 IgM was purified with a low yield when using Rhinophase-AB, we used porous zirconia particles in the column for the purification of PA5 IgM only.

### ELISA

ELISA was performed as described previously^9,29,30^. For the analysis of glycoconjugates, 500 ng of glycosphingolipids dissolved in methanol, or 1 µg of fetuin dissolved in distilled water was added to the wells of a 96-well microtiter plate then incubated for fixation onto the plate. For the analysis of ssDNA and dsDNA, the wells of the 96-well microtiter plate were pre-coated with a 0.0125% of ε-Poly-l-lysine coating solution (COSMO BIO, Tokyo, Japan), and 250 ng of ssDNA or dsDNA dissolved in distilled water was added for fixation of the DNA onto the plate. The ssDNA was prepared by boiling at 100 °C for 10 min, then rapidly cooling on ice. After washing twice with phosphate-buffered saline (PBS), blocking buffer (1% BSA in PBS) was added to each well, and incubated for 15 min at room temperature, followed by the addition of diluted antibodies. After incubation for 2.5 h at room temperature, the wells were washed with 0.05% Tween 20 in PBS, then horseradish peroxidase (HRP)-linked secondary antibody (anti-IgM or anti-IgG) was added. Antibody binding was detected using an HRP substrate (1-Step Ultra TMB-ELISA Substrate; Thermo Fisher Scientific), and the absorbance was measured at 450 nm. Samples were analyzed in duplicate in each experiment, and two independent experiments were performed for all experiments.

### SEC-HPLC analysis

SEC-HPLC analyses of zirconia column chromatography-purified samples were performed with an HPLC system (LC-2000, JASCO, Tokyo, Japan) using a TSKgel UltraSW Aggregate column (300 mm × 7.8 mm in diameter; particle size, 3 μm; Tosoh, Tokyo, Japan) as described previously^17,18^. Chromatographic analysis was carried out at 25 °C on the column using 0.2 M phosphate buffer (pH 6.7) as the mobile phase at a flow rate of 0.8 ml/min in the isocratic mode. Immunoglobulins and other proteins were detected using an in-line ultraviolet detector at 280 nm.

### Flow cytometric analysis

The expression of glycan epitopes on the cell surface was analyzed using an RF-500 flow cytometer (Sysmex, Tokyo, Japan). HUVECs purchased from KURABO (Osaka, Japan) were maintained in HuMedia-EG2 (KURABO) at 37 °C in a humidified atmosphere containing 5% CO_2_. The HUVECs have been obtained under proper informed consent and adheres to the Declaration of Helsinki, the Human Tissue Act (UK), Title 21 of the Code of Federal Regulations (USA), and HIPAA regulations relative to obtaining and handling human tissue for research use. 1 × 10^6^ cells were seeded onto a culture dish (100 mm in diameter) and incubated overnight. After the incubation, cells were harvested using 5 mM ethylenediaminetetraacetic acid in PBS, and suspended in 200 μl of cold PBS. The suspensions were incubated with 0.2 μg of FR9, AFR45, or PA5 monoclonal IgM on ice, then sequentially labeled with Alexa 488-conjugated anti-mouse IgM antibody (Thermo Fisher Scientific).

### Supplementary Information

Supplementary Information.

## Data Availability

The datasets generated during and/or analyzed during the current study are available from the corresponding author on reasonable request.

## References

[1] Storry JR, Olsson ML (2009). The ABO blood group system revisited: A review and update. Immunohematology.

[2] Temme JS, Butler DL, Gildersleeve JC (2021). Anti-glycan antibodies: Roles in human disease. Biochem. J..

[3] Koike H, Chiba A, Katsuno M (2021). Emerging infection, vaccination, and Guillain–Barré syndrome: A review. Neurol. Ther..

[4] Pisetsky DS, Lipsky PE (2020). New insights into the role of antinuclear antibodies in systemic lupus erythematosus. Nat. Rev. Rheumatol..

[5] Egner W (2000). The use of laboratory tests in the diagnosis of SLE. J. Clin. Pathol..

[6] Gillmann KM, Temme JS, Marglous S, Brown CE, Gildersleeve JC (2023). Anti-glycan monoclonal antibodies: Basic research and clinical applications. Curr. Opin. Chem. Biol..

[7] Okuda T (2021). Application of the antibody-inducing activity of glycosphingolipids to human diseases. Int. J. Mol. Sci..

[8] Okuda T, Fukui A (2018). Generation of anti-oligosaccharide antibodies that recognize mammalian glycoproteins by immunization with a novel artificial glycosphingolipid. Biochem. Biophys. Res. Commun..

[9] Okuda T, Shimizu K, Hasaba S, Date M (2019). Induction of specific adaptive immune responses by immunization with newly designed artificial glycosphingolipids. Sci. Rep..

[10] Okuda T, Kato K (2022). Glycosphingolipids form characteristic-sized liposomes that correlate with their antibody-inducing activities in mice. Biochem. Biophys. Res. Commun..

[11] Okuda T, Kitamara M, Kasahara S, Kato K (2021). Identification of genes for variable regions of immunoglobulins that recognize sialylated glycans. Biochem. Biophys. Res. Commun..

[12] Okuda T (2017). PUGNAc treatment provokes globotetraosylceramide accumulation in human umbilical vein endothelial cells. Biochem. Biophys. Res. Commun..

[13] Okuda T (2020). Isolation and characterization of antibodies induced by immunization with TNF-α inducible globotetraosylceramide. Int. J. Mol. Sci..

[14] Kubota T, Akatsuka T, Kanai Y (1986). A monoclonal anti-double stranded DNA antibody from an autoimmune MRL/Mp-lpr/lpr mouse: Specificity and idiotype in serum immunoglobulins. Immunol. Lett..

[15] Krishnan MR, Jou NT, Marion TN (1996). Correlation between the amino acid position of arginine in VH-CDR3 and specificity for native DNA among autoimmune antibodies. J. Immunol..

[16] Wloch MK, Alexander AL, Pippen AM, Pisetsky DS, Gilkeson GS (1997). Molecular properties of anti-DNA induced in preautoimmune NZB/W mice by immunization with bacterial DNA. J. Immunol..

[17] Okuda T, Kato K, Kitamura M, Kasahara S (2021). Purification of anti-glycoconjugate monoclonal antibodies using newly developed porous zirconia particles. Sci. Rep..

[18] Okuda T, Kitamura M, Kato K (2022). A zirconia-based column chromatography system optimized for the purification of IgM from hybridoma culture supernatants. Anal. Biochem..

[19] Blank M, Barzilai O, Shoenfeld Y (2007). Molecular mimicry and auto-immunity. Clin. Rev. Allergy Immunol..

[20] Hanasaki K, Varki A, Stamenkovic I, Bevilacqua MP (1994). Cytokine-induced β-galactoside α-2,6-sialyltransferase in human endothelial cells mediates α2,6-sialylation of adhesion molecules and CD22 ligands. J. Biol. Chem..

[21] Okuda T (2006). Targeted disruption of Gb3/CD77 synthase gene resulted in the complete deletion of globo-series glycosphingolipids and loss of sensitivity to verotoxins. J. Biol. Chem..

[22] Okuda T, Nakakita S, Nakayama K (2010). Structural characterization and dynamics of globotetraosylceramide in vascular endothelial cells under TNF-α stimulation. Glycoconj. J..

[23] Okuda T (2018). Data set for characterization of TNF-α-inducible glycosphingolipids in vascular endothelial cells. Data Brief.

[24] Kim SJ (2014). Monosialic ganglioside GM3 specifically suppresses the monocyte adhesion to endothelial cells for inflammation. Int. J. Biochem. Cell Biol..

[25] Gunji M (2023). Gemcitabine alters sialic acid binding of the glycocalyx and induces inflammatory cytokine production in cultured endothelial cells. Med. Mol. Morphol..

[26] Calle-Botero E, Abril A (2020). Lupus vasculitis. Curr. Rheumatol. Rep..

[27] Percie du Sert N (2020). The ARRIVE guidelines 2.0: Updated guidelines for reporting animal research. PLoS Biol..

[28] Green ED, Adelt G, Baenziger JU, Wilson S, Van Halbeek H (1988). The asparagine-linked oligosaccharides on bovine fetuin: Structural analysis of N-glycanase-released oligosaccharides by 500-megahertz H NMR spectroscopy. J. Biol. Chem..

[29] Aotsuka S, Okawa M, Ikebe K, Yokohari R (1979). Measurement of anti-double-stranded DNA antibodies in major immunoglobulin classes. J. Immunol. Methods.

[30] Zouali M, Stollar BD (1986). A rapid ELISA for measurement of antibodies to nucleic acid antigens using UV-treated polystyrene microplates. J. Immunol. Methods.

