# Supplementary material for: Serum components influence antibody reactivity to glycan and DNA antigens
Source: Sci Rep. 2023 Aug 22;13:13644. doi: 10.1038/s41598-023-40707-3 (PMC10444758; doi:10.1038/s41598-023-40707-3)
Supplement: Supplementary file 1 — Supplementary Information. [file 41598_2023_40707_MOESM1_ESM.pdf]

Serum components influence antibody reactivity to glycan and DNA antigens

Tetsuya Okuda[1] & Katsuya Kato[2]

1. Bioproduction Research Institute, National Institute of Advanced Industrial Science and Technology (AIST), Central6, 1-1-1 Higashi, Tsukuba 305-8566, Japan.

2. Multi-Material Research Institute, National Institute of Advanced Industrial Science and Technology (AIST), 2266-98 Anagahora, Shimoshidami, Moriyama-ku, Nagoya 463-8560, Japan.

Supplementary Information

Supplemental Table S1
Supplemental Table S2
Supplemental Figure S1
Supplemental Figure S2
Supplemental Figure S3
Supplemental Figure S4

Clone ID/Accession No.	Query Cover (%)	Per. Indent (%)	Class	Strain
FR9-V_H vs.				
202.135/Z22119	100	95.2	IgM	NZB/W F1
111.109/Z22022	100	95.2	IgG	NZB/W F1
202.61/Z22127	100	94.7	IgM	NZB/W F1
423s.92/U55526	97	94.3	IgG	NZB/W F1
F1-3/U60455	83	99.3	IgM	NZB/W F1
AFR45-V_H vs.				
MRL-DNA22/M20835	100	95.5	IgM	MRL/lpr
111.185vh/Z22024	84	100	IgM	NZB/W F1
165.27/Z22053	84	98.3	IgM	NZB/W F1
2C10/U23046	84	98.0	IgG	MRL/lpr
165.49/Z22064	78	100	IgG	NZB/W F1
FR9/AFR45-V_L vs.				
B.203/AF072796	94	97.7	IgK	BALB/c
B.21/AF072789	96	96.4	IgK	BALB/c
452p.53/U55663	94	97.0	IgK	NZB/W F1
PA5-V_H vs.				
ZA5E11/AY436920	97	96.8	IgM	NZM2410
163.100/Z22036	85	99.7	IgM	NZB/W F1
363p.168/U55447	79	100	IgM	NZB/W F1
PA5-V_L vs.				
F4-2/U60466	98	97.1	IgK	NZB/W F1
202.61/Z22128	96	97.7	IgK	NZB/W F1
84.26/U30631	89	99.7	IgK	NZB/W F1

Supplementary Table S1. **Genes encoding anti-DNA antibodies with high homology in the nucleotide sequences for variable regions to those in the anti-glycan IgM genes.** Comparisons of the sequences with the highest homology for each variable region are shown in Fig. S1.

Abbreviation	Structure
6SLN	Siaα2,6Galβ1,4GlcNAcβ1,1CerA
3SLN	Siaα2,3Galβ1,4GlcNAcβ1,1CerA
LacNAc	Galβ1,4GlcNAcβ1,1CerA
Gb4	GalNAcβ1,3Galα1,4Galβ1,4Glcβ1,1Cer
Gb3	Galα1,4Galβ1,4Glcβ1,1Cer
LacCer	Galβ1,4Glcβ1,1Cer
GM3	Siaα2,3Galβ1,4Glcβ1,1Cer
GD3	Siaα2,8Siaα2,3Galβ1,4Glcβ1,1Cer
GD1a	Siaα2,3Galβ1,3GalNAcβ1,4(Siaα2,3)Galβ1,4Glcβ1,1Cer
GT1b	Siaα2,3Galβ1,3GalNAcβ1,4(Siaα2,8Siaα2,3)Galβ1,4Glcβ1,1Cer
GM1	Galβ1,3GalNAcβ1,4(Siaα2,3)Galβ1,4Glcβ1,1Cer
GM2	GalNAcβ1,4(Siaα2,3)Galβ1,4Glcβ1,1Cer
GD2	GalNAcβ1,4(Siaα2,8Siaα2,3)Galβ1,4Glcβ1,1Cer
GA2	GalNAcβ1,4Galβ1,4Glcβ1,1Cer
GA1	Galβ1,3GalNAcβ1,4Galβ1,4Glcβ1,1Cer
Fetuin*	Siaα2,3(6)Galβ1,4GlcNAc
Fetuin-a*	Galβ1,4GlcNAc
Fetuin-b*	Siaα2,6Galβ1,4GlcNAc

Supplementary Table S2. **Oligosaccharide structures of the glycoconjugates used in this study.** *The main structures of the non-reducing terminal oligosaccharides in these glycoproteins are shown. Abbreviations: Cer, ceramide; Sia, sialic acid (Neu5Ac); CerA, ceramide analogue; Fetuin-a, asialofetuin; Fetuin-b, α2,3-sialidase-treated fetuin.

```
FR9 VH      1  GAGGTGCAGCTTCAGCAGTCTGGACCTGAGCTGGTAAAGCCTGGGGCTTCAGTGAAGATG  60
               ***** ***** ************************************************
202.135     1  GAGGTCCAGCTGCAGCAGTCTGGACCTGAGCTGGTAAAGCCTGGGGCTTCAGTGAAGATG  60

FR9 VH     61  TCCTGCAAGGCTTCTGGATACACATTCACTAGCTATGTTATGCACTGGGTGAAGCAGAAG  120
               ************************************************************
202.135    61  TCCTGCAAGGCTTCTGGATACACATTCACTAGCTATGTTATGCACTGGGTGAAGCAGAAG  120

FR9 VH    121  CCTGGGCAGGGCCTTGAGTGGATTGGATATATTAATCCTTACAATGATGGTACTAAGTAC  180
               ************************************************************
202.135   121  CCTGGGCAGGGCCTTGAGTGGATTGGATATATTAATCCTTACAATGATGGTACTAAGTAC  180

FR9 VH    181  AATGAGAAGTTCAAAGGCAAGGCCACACTGACTTCAGACAAATCCTCCAGCACAGCCTAC  240
               ************************************************************
202.135   181  AATGAGAAGTTCAAAGGCAAGGCCACACTGACTTCAGACAAATCCTCCAGCACAGCCTAC  240

FR9 VH    241  ATGGAGCTCAGCAGCCTGACCTCTGAGGACTCTGCGGTCTATTACTGTGC----------  290
               **************************************************
202.135   241  ATGGAGCTCAGCAGCCTGACCTCTGAGGACTCTGCGGTCTATTACTGTGCAAGAGGTTAC  300

FR9 VH    291  -----GAGTAGTAATACCTGGTTTGCTTACTGGGGCCAAGGGACTCTGGTCACTGTCTCT  345
                     *****  *  ********************************************
202.135   301  TACGGTAGTAGCTACTCCTGGTTTGCTTACTGGGGCCAAGGGACTCTGGTCACTGTCTCT  360

FR9 VH    346  GCA  348
               ***
202.135   361  GCA  363
```

```
PA5 VH     10  CTGCAGCAGCCTGGGGCTGAGCTTGTGAAGCCTGGGGCTTCAGTGAAGTTGTCCTGCAAG  69
               ********* * ************************************************
ZA5E11      1  CTGCAGCAGTCAGGGGCTGAGCTTGTGAAGCCTGGGGCTTCAGTGAAGTTGTCCTGCAAG  60

PA5 VH     70  GCTTCTGGCTACACCTTCACCAGCTACTGGATGCACTGGGTGAAGCAGAGGCCTGGACAA  129
               ************************************************************
ZA5E11     61  GCTTCTGGCTACACCTTCACCAGCTACTGGATGCACTGGGTGAAGCAGAGGCCTGGACAA  120

PA5 VH    130  GGCCTTGAGTGGATTGGAGAGATTAATCCTAGCAATGGTGGTACTAACTACAATGAGAAG  189
               ************************************************************
ZA5E11    121  GGCCTTGAGTGGATTGGAGAGATTAATCCTAGCAATGGTGGTACTAACTACAATGAGAAG  180

PA5 VH    190  TTCAAGAGCAAGGCCACACTGACTGTAGACAAATCCTCCAGCACAGCCTACATGCAACTC  249
               ************************************************************
ZA5E11    181  TTCAAGAGCAAGGCCACACTGACTGTAGACAAATCCTCCAGCACAGCCTACATGCAACTC  240

PA5 VH    250  AGCAGCCTGACATCTGAGGACTCTGCGGTCTATTACTGTAC------AACTTGGTACTTC  303
               *****************************************       *  *********
ZA5E11    241  AGCAGCCTGACATCTGAGGACTCTGCGGTCTATTACTGTACGGGGGGTAGGTGGTACTTC  300

PA5 VH    304  GATGTCTGGGGCGCAGGGACCACGGTCACCGTCTCCTCA  342
               ***************************************
ZA5E11    301  GATGTCTGGGGCGCAGGGACCACGGTCACCGTCTCCTCA  339
```

```
AFR45 VH     1  CAGGTCCAACTGCAGCAGCCTGGTGCTGAGCTTGTGAAGCCTGGGGCCTCAGTGAAGCTG  60
                ************************************************************
MRL-DNA22  106  CAGGTCCAACTGCAGCAGCCTGGTGCTGAGCTTGTGAAGCCTGGGGCCTCAGTGAAGCTG  165

QAFR45 VH   61  TCCTGCAAGGCTTCTGGCTACACTTTCACCAGCTACTGGATAAACTGGGTGAAGCAGAGG  120
                ************************************************************
MRL-DNA22  166  TCCTGCAAGGCTTCTGGCTACACTTTCACCAGCTACTGGATAAACTGGGTGAAGCAGAGG  225

AFR45 VH   121  CCTGGACAAGGCCTTGAGTGGATTGGAAATATTTATCCTGGTAGTAGTAGTACTAACTAC  180
                ************************************************************
MRL-DNA22  226  CCTGGACAAGGCCTTGAGTGGATTGGAAATATTTATCCTGGTAGTAGTAGTACTAACTAC  285

AFR45 VH   181  AATGAGAAGTTCAAGAGCAAGGCCACACTGACTGTAGACACATCCTCCAGCACAGCCTAC  240
                ************************************************************
MRL-DNA22  286  AATGAGAAGTTCAAGAGCAAGGCCACACTGACTGTAGACACATCCTCCAGCACAGCCTAC  345

AFR45 VH   241  ATGCAGCTCAGCAGCCTGACATCTGACGACTCTGCGGTCTATTATTGTGCAAGA-GCC--  297
                ****************************************************** ***
MRL-DNA22  346  ATGCAGCTCAGCAGCCTGACATCTGACGACTCTGCGGTCTATTATTGTGCAAGACGCCTA  405

AFR45 VH   298  TATAGTAACT------GGGACTACTGGGGCCAAGGCACCACTCTCACAGTCTCCTCA  348
                *****  ***       ************ ***** *** *  **** *********
MRL-DNA22  406  TATAGGTACTATGCTATGGACTACTGGGGTCAAGGAACCTCAGTCACCGTCTCCTCA  462
```

```
PA5 VL      1  GACAATGTTCTCACCCAGTCTCCAGCAATCATGTCTGCATCTCCAGGGGAGAAGGTCACC  60
               * * *******************************************************
F4-2        1  CAAATTGTTCTCACCCAGTCTCCAGCAATCATGTCTGCATCTCCAGGGGAGAAGGTCACC  60

PA5 VL     61  ATGACCTGCAGTGCCAGCTCAAGTGTAAGTTACATGCACTGGTACCAGCAGAAGTCAGGC  120
               ************************************************************
F4-2       61  ATGACCTGCAGTGCCAGCTCAAGTGTAAGTTACATGCACTGGTACCAGCAGAAGTCAGGC  120

PA5 VL    121  ACCTCCCCCAAAAGATGGATTTATGACACATCCAAACTGGCTTCTGGAGTCCCTGCTCGC  180
               ************************************************************
F4-2      121  ACCTCCCCCAAAAGATGGATTTATGACACATCCAAACTGGCTTCTGGAGTCCCTGCTCGC  180

PA5 VL    181  TTCAGTGGCAGTGGGTCTGGGACCTCTTACTCTCTCACAATCAGCAGCATGGAGGCTGAA  240
               ************************************************************
F4-2      181  TTCAGTGGCAGTGGGTCTGGGACCTCTTACTCTCTCACAATCAGCAGCATGGAGGCTGAA  240

PA5 VL    241  GATGCTGCCACTTATTACTGCCAGCAGTGGAGTAGTAACCCGTGGACGTTCGGTGGAGGC  300
               ************************************.****    ******** ** **
F4-2      241  GATGCTGCCACTTATTACTGCCAGCAGTGGAGTAGTNACCCACCCACGTTCGGAGGGGGG  300

PA5 VL    301  ACCAAGCTGGAAATCAAA  318
               ************** ***
F4-2      301  ACCAAGCTGGAAATAAAA  318
```

```
FR9/AFR45 VL    6  TGTTCTCACCCAGTCTCCAGCAATCATGTCTGCATCTCCAGGGGAGAAGGTCACCATAAC  65
                   ************************************************************
B.203           1  TGTTCTCACCCAGTCTCCAGCAATCATGTCTGCATCTCCAGGGGAGAAGGTCACCATAAC  60

FR9/AFR45 VL   66  CTGCAGTGCCAGCTCAAGTGTAAGTTACATGCACTGGTTCCAGCAGAAGCCAGGCACTTC  125
                   ***********************************.*******..***************
B.203          61  CTGCAGTGCCAGCTCAAGTGTAAGTTACATGCACTNGTTCCAGNNGAAGCCAGGCACTTC  120

FR9/AFR45 VL  126  TCCCAAACTCTGGATTTATAGCACATCCAACCTGGCTTCTGGAGTCCCTGCTCGCTTCAG  185
                   ************************************************************
SB.203        121  TCCCAAACTCTGGATTTATAGCACATCCAACCTGGCTTCTGGAGTCCCTGCTCGCTTCAG  180

FR9/AFR45 VL  186  TGGCAGTGGATCTGGGACCTCTTACTCTCTCACAATCAGCCGAATGGAGGCTGAAGATGC  245
                   ******************************.*********************.*******
B.203         181  TGGCAGTGGATCTGGGACCTCTTACTCTCTNACAATCAGCCGAATGGAGGCTRAAGATGC  240

FR9/AFR45 VL  246  TGCCACTTATTACTGCCAGCAAAGGAGTAGTTACCCATTCACGTTCGGCTCGGGGACAAA  305
                   *******************.*************** ************************
B.203         241  TGCCACTTATTACTGCCAGNAAAGGAGTAGTTACCAATTCACGTTCGGCTCGGGGACAAA  300

FR9/AFR45 VL  306  G  306
                   *
B.203         301  G  301
```

Supplementary Figure S1. Homology analysis of the nucleotide sequences for anti-glycan and anti-DNA antibodies. Asterisks indicate conserved nucleotides.

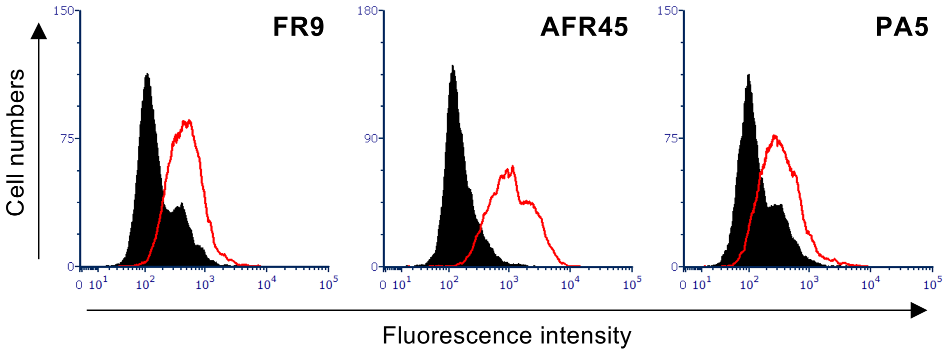

Supplementary Figure S2. Flow cytometric analysis of anti-glycan IgM antibody epitopes on the surface of HUVECs.

HUVECs were stained with 2 μg/ml of FR9 (left panel), AFR45 (middle panel), or PA5 (right panel) as the primary antibody, then with Alexa 488-labeled secondary antibody (red lines). Controls for background monitoring were prepared using a standard mouse IgM and the secondary antibody (dark shading).

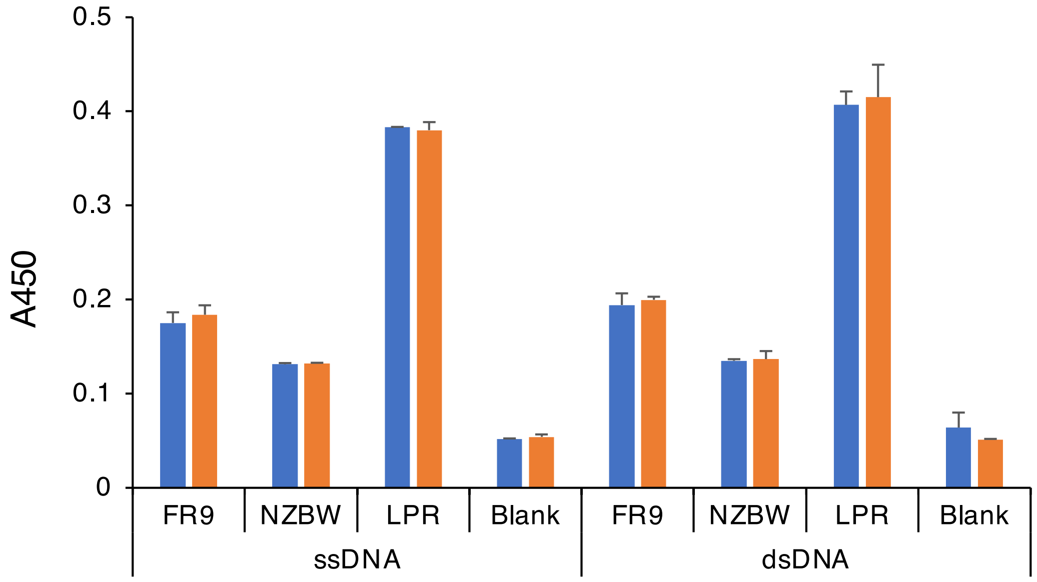

Supplementary Figure S3. Analysis of the inhibitory effect of fetuin on the binding of FR9 IgM and serum immunoglobulins to DNA.

Purified immunoglobulins (2 μg/ml) from FR9 hybridoma culture supernatant (FR9), and NZB/W F1 (NZBW) and MRL/lpr (LPR) mouse sera were pre-incubated with 2 μg/ml of fetuin, then applied to ssDNA-coated or dsDNA-coated microplate wells for the determination of reactivity by ELISA (orange bars). The reactivity of purified immunoglobulins that were not pretreated with fetuin is shown as a control (blue bars). Error bars indicate the mean ± standard deviation (n = 4).

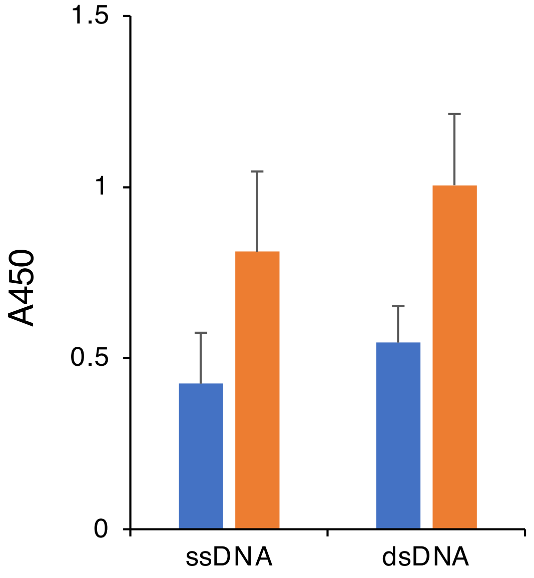

Supplementary Figure S4. Analysis of the inhibitory effect of dsDNA on the binding of FR9 IgM to DNA.

Purified immunoglobulins (2 μg/ml) from FR9 hybridoma culture supernatant were pre-incubated with 5 μg/ml of dsDNA, then applied to ssDNA-coated or dsDNA-coated microplate wells for the determination of reactivity by ELISA (orange bars). The reactivity of purified immunoglobulins that were not pretreated with dsDNA is shown as a control (blue bars). Error bars indicate the mean ± standard deviation (n = 4).
